# The first complete chloroplast genome sequence of *Paris polyphylla* var. *emeiensis,* a rare and endangered species

**DOI:** 10.1080/23802359.2020.1768942

**Published:** 2020-05-27

**Authors:** Min Fan, Jie Liu, Jing Wang, Jun Qian, Conglong Xia, Baozhong Duan

**Affiliations:** a College of Pharmaceutical Science, Dali University, Dali, China; b Key Laboratory of Yunnan Provincial Higher Education Institute for Development of Yunnan Daodi Medicinal Materials Resources, Dali, China

**Keywords:** *Paris polyphylla* var. *emeiensis*, complete chloroplast genome, phylogeny, Melanthiaceae

## Abstract

*Paris polyphylla* var. *emeiensis* H. X. Yin, H. Zhang & D. Xue is a member of the genus *Paris* endemic to southwest of China. The complete chloroplast (cp) genome of *P. polyphylla* var. *emeiensis* was 164,854 bp in length with 36.96% overall GC content, including a large single-copy (LSC) region of 84,438 bp and a small single-copy (SSC) region of 12,892 bp, which were separated by a pair of inverted repeats (IRs) of 33,762 bp. There were 135 genes in total, including 89 protein-coding genes, 38 tRNA genes, and 8 rRNA genes. Phylogenetic analysis indicated *P. polyphylla* var. *emeiensis* was closely related to *P. fargesii* and *P. cronquistii.*

*Paris polyphylla* var. *emeiensis*, an endemic to southwest of China, is a species of flowering herb of the family Melanthiaceae. This species was described from Mount Emei, Sichuan Province, China in 2007 for the first time (Yin et al. 2007). The root of *P. polyphylla* var. *emeiensis* was used as the substitute of the traditional Chinese medicine ‘Chonglou’, which have been used for the treatment of hemostasis, sore throat, parotitis, furuncle, carbuncle, snake bite, and convulsion (Fu et al. 2012; Liu et al. 2017; Duan et al. 2018). However, little genetic information is known about this species until now. Herein, we assembled and characterized the complete chloroplast (cp) genome of *P. polyphylla* var. *emeiensis* for the first time, and its phylogenetic analysis was investigated. The study will provide useful information for the conservation and utilization of this species.

Fresh and clean leaves of *P. polyphylla* var. *emeiensis* were sampled from Gucheng District, Lijiang, Yunnan, China (26°74′71″N, 100°27′14″E). Meanwhile, a voucher specimen was collected and deposited at the Herbarium of Dali University (20191003B2). The total DNA was extracted using the DNeasy plant mini kit (QIAGEN), and sequenced by Illumina NovaSeq system (Illumina, San Diego, CA, USA). About 7.30 Gb of raw data (51, 901, 266 reads) were assembled by NOVOPlasty (Park et al. 2019; Liu et al. 2020), and the assembled cp genome was annotated by GeSeq with default sets (Tillich et al. 2017; Qian et al. 2020). The annotated cp genome was submitted to the GenBank with the accession number of MT381945.

The cp genome of *P. polyphylla* var. *emeiensis* was 164,854 bp in length with a typical quadripartite structure of angiosperms, containing a large single-copy (LSC) region of 84,438 bp, a small single-copy (SSC) region of 12,892 bp, and a pair of inverted repeats (IRs) region of 33,762 bp. The genome comprises of 135 genes, including 89 protein-coding genes, 38 tRNA genes, and 8 ribosomal RNA genes. The overall GC content was 36.96%. To confirm the phylogenetic relationship of *P. polyphylla* var. *emeiensis*, a maximum likelihood (ML) bootstraps analysis was performed based on 25 cp genomes. The sequences were aligned by MAFFT v7.307 (Katoh and Standley 2013; Jiang et al. 2020), and the phylogenetic tree was constructed by RAxML (Stamatakis 2014), with *Dioscorea aspersa* (NC 039807) and *Dioscorea baya* (NC 039850) as outgroups. Phylogenetic analysis showed that *P. polyphylla* var. *emeiensis* was closely related to *P. fargesii* and *P. cronquistii* (Figure 1). The cp genome of *P. polyphylla* var. *emeiensis* will provide a useful resource for the conservation genetics of this species as well as for phylogenetic studies of *Paris* genus.

**Figure 1.**
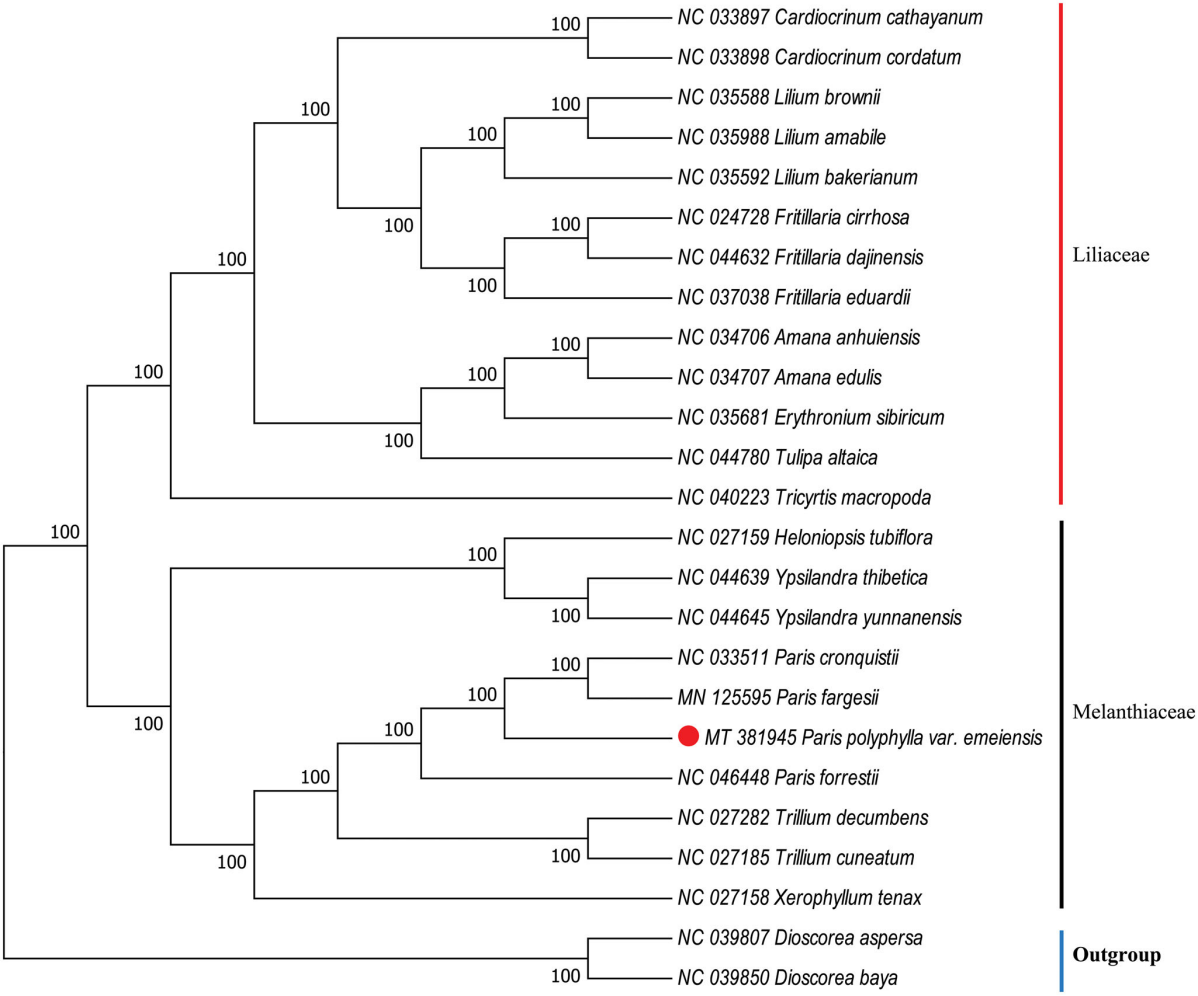
Phylogenetic analysis of 22 species and two taxa as outgroups based on chloroplast genome sequences by RAxML, bootstrap support value near the branch.

## Data Availability

The data that support the findings of this study are openly available in NCBI GenBank database at (https://www.ncbi.nlm.nih.gov) with the accession number is MT381945, which permits unrestricted use, distribution, and reproduction in any medium, provided the original work is properly cited.
